# BIFI: a Taverna plugin for a simplified and user-friendly workflow platform

**DOI:** 10.1186/1756-0500-7-740

**Published:** 2014-10-20

**Authors:** Ahmet Yildiz, Erkan Dilaveroglu, Ilhami Visne, Bilal Günay, Emrah Sefer, Andreas Weinhausel, Frank Rattay, Carole A Goble, Ram Vinay Pandey, Albert Kriegner

**Affiliations:** Health & Environment Department, AIT Austrian Institute of Technology GmbH, Vienna, Austria; Institute for Analysis and Scientific Computing, Vienna University of Technology, 1040 Vienna, Austria; Department of computer Science, University of Manchester, Oxford Road, Manchester, M13 9PT UK; Institut für Populationsgenetik, Vetmeduni Vienna, Veterinärplatz 1, A-1210 Vienna, Austria

## Abstract

**Background:**

Heterogeneity in the features, input-output behaviour and user interface for available bioinformatics tools and services is still a bottleneck for both expert and non-expert users. Advancement in providing common interfaces over such tools and services are gaining interest among researchers. However, the lack of (meta-) information about input-output data and parameter prevents to provide automated and standardized solutions, which can assist users in setting the appropriate parameters. These limitations must be resolved especially in the workflow-based solution in order to ease the integration of software.

**Findings:**

We report a Taverna Workbench plugin: the XworX BIFI (*Beautiful Interfaces for Inputs*) implemented as a solution for the aforementioned issues. BIFI provides a *Graphical User Interface* (GUI) definition language used to layout the user interface and to define parameter options for Taverna workflows. BIFI is also able to submit *GUI Definition Files* (GDF) directly or discover appropriate instances from a configured repository. In the absence of a GDF, BIFI generates a default interface.

**Conclusion:**

The Taverna Workbench is an open source software providing the ability to combine various services within a workflow. Nevertheless, users can supply input data to the workflow via a simple user interface providing only a text area to enter the input in text form. The workflow may contain meta-information in human readable form such as description text for the port and an example value. However, not all workflow ports are documented so well or have all the required information.

BIFI uses custom user interface components for ports which give users feedback on the parameter data type or structure to be used for service execution and enables client-side data validations. Moreover, BIFI offers user interfaces that allow users to interactively construct workflow views and share them with the community, thus significantly increasing usability of heterogeneous, distributed service consumption.

**Electronic supplementary material:**

The online version of this article (doi:10.1186/1756-0500-7-740) contains supplementary material, which is available to authorized users.

## Findings

### Background

The growing need for complex *in-silico* experiments on bioinformatics data leads to applications which are able to provide dynamic interfaces for automated analysis: workflow based software. Workflows are pre-defined series of tasks that are related to each other by the flow of data between them [1]. Workflow based systems are able to use various services deployed either on the local machine or on a remote device. Remote services utilized in Taverna [2] are usually deployed using SOAP based web services which are characterized by their great interoperability and extensibility, as well as their machine-processable descriptions based on XML [3]. The description of web services in WSDL (*Web Service Description Language*) format enables the description of the abstract functionality offered by a service to be separated from concrete details of a service description such as “how” and “where” that functionality is offered [4]. This abstraction makes the platform used to deploy the services perfectly independent of the platform utilizing the service. Besides consuming SOAP based web services, Taverna supports Restful, BioMoby [5] and Soaplab [6] services. Moreover, it can locally utilize Java code snippets, Beanshell [7] scripts and R scripts (through RShell [8]).

The Taverna Workbench is a fully featured, extensible and scalable scientific workflow management system [2] built by The ^my^Grid Team [9]. It has access to over 3,500 local and remote resources and analysis tools, web and grid services available on start-up [10].

### The current scenario in Taverna

**Figure 1:**
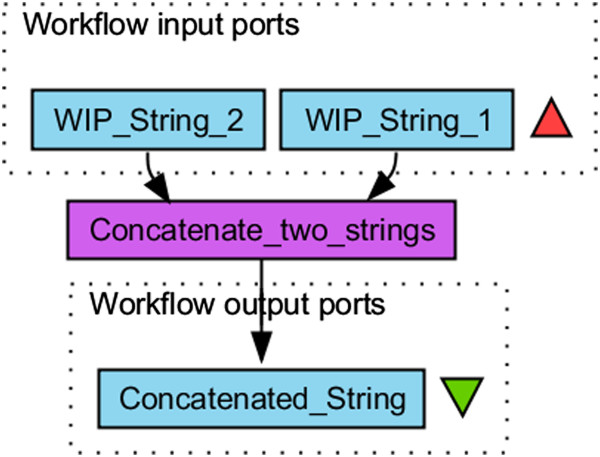
**A simple Taverna workflow.** The Graph of a simple workflow from The Taverna Workbench consisting of two workflow input ports, one processor and one workflow output port.

A simple workflow in Taverna consists of one or more *Workflow Input Ports* (WIP), a processor, itself consisting of input and output ports, executing the actual logic and an output port collecting the resulting analysis data. See Figure 1 for a simple workflow graph.

The creation (design) and the execution of the workflow are two independent steps. After designing a workflow, users specify data for the WIPs, which are then routed to the next processor during the execution time.

**Figure 2:**
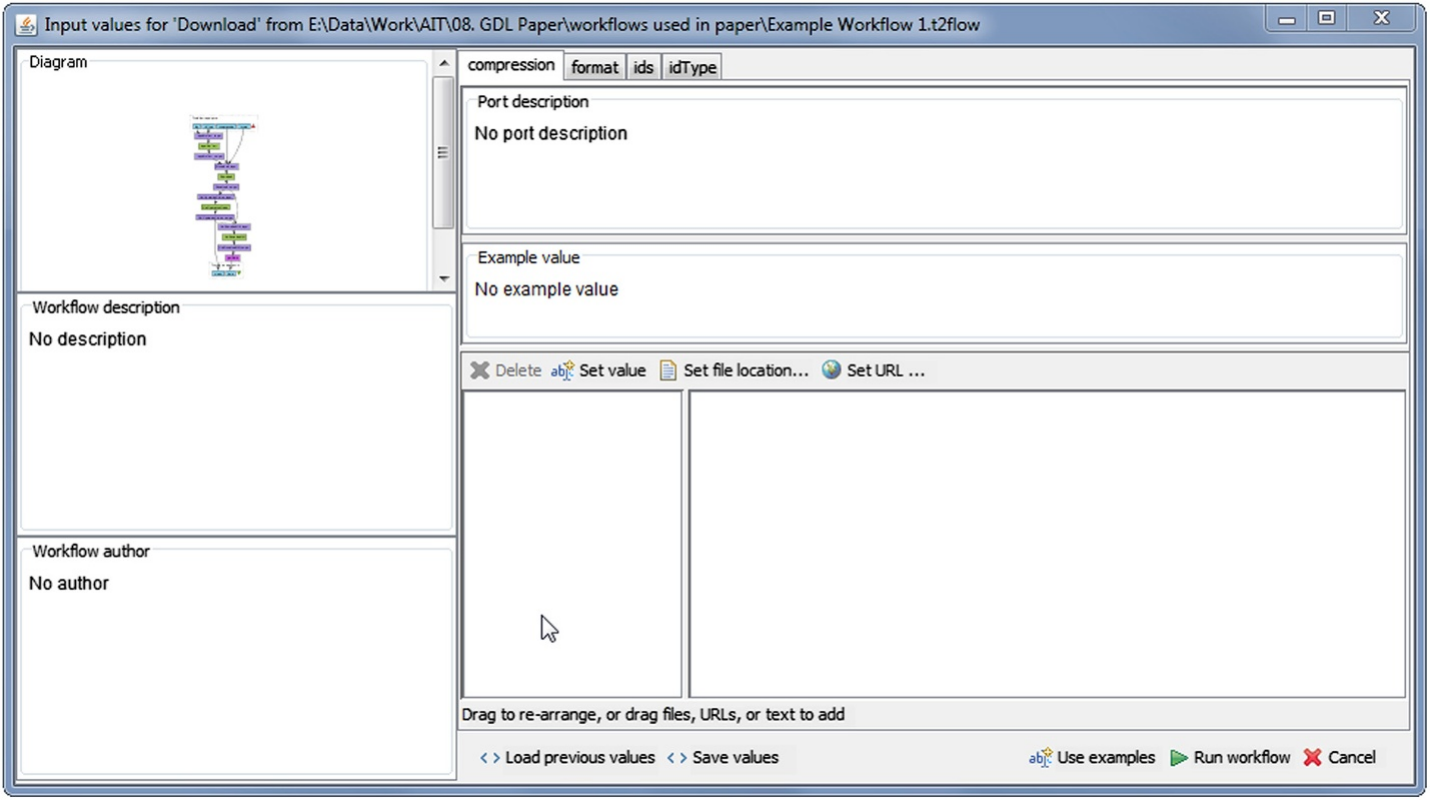
**Taverna dialog for WIP.** A screenshot of the Taverna workbench shows the input dialog to enter the data for WIPs. It provides only a text area to enter the input in text form.

WIPs carry no metadata in a computer interpretable form, however, except information about the data dimension. But this information is apparently not sufficient to provide the advanced, self-describing user interfaces, which give a sense of the type and format of the data to be entered for analysis. Figure 2 shows the Taverna dialog for setting input values of WIPs generated for the *Example Workflow 1 as* supplied as Additional file 1. This dialog provides users only with text areas to enter the input data in text form. This limitation is due partly to a shortage of remote services provided by autonomous third parties around the world having insufficient or non-existent metadata. Examining SOAP based web services, which constitute the majority of remote services listed in Taverna, the metadata are often as simple as data type definitions like ‘string’ which have no information about complex file formats [2]. This shortcoming may make the data type so unpredictable that users should basically execute the service using some data and see what happens. Erroneous results can be expected which could easily lead to disappointment and deter users from using the system [11].

### Solution projection

Enabling resource consumption from various local and remote sources brings great advantage in scalability, but also increases complexity in software development and use. This complexity not only decreases the usability level but also increases training times required to get used to the software.

We aim to provide interfaces that are intelligent enough to support users in entering the right data required by the service and thus improve the system’s trustworthiness and predictability.

**Figure 3:**
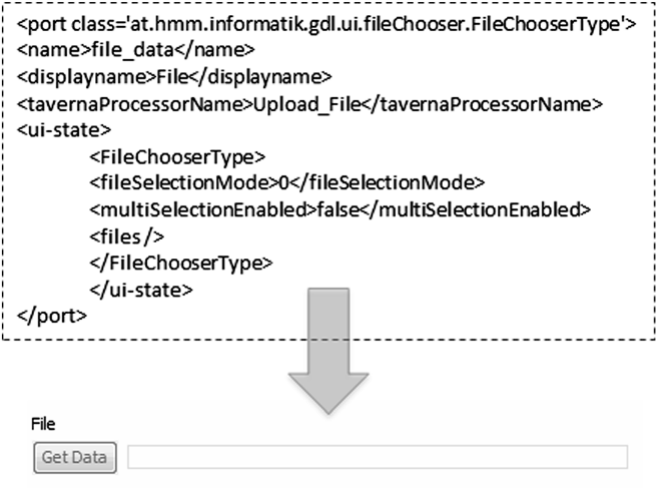
**An example port definition and generated UI.** An outline of a port definition and the generated user interface component using it.

**Figure 4:**
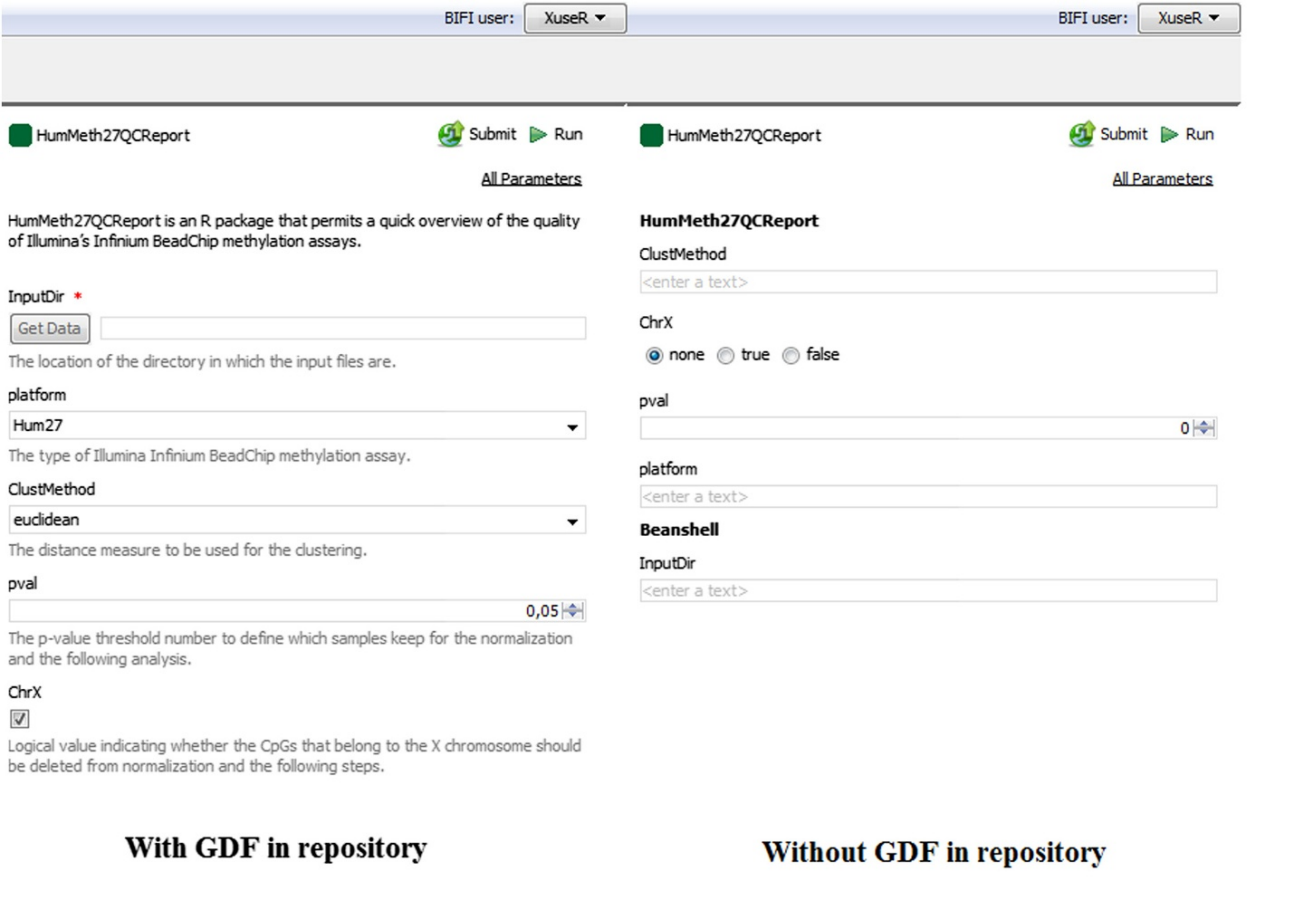
**Comparison of example workflow 2 opened with and without using GDF.** Example Workflow 2 consists of an R script with 5 input ports. BIFI is able to generate user interfaces for two of the ports automatically (right) if no GDF is found in the repository. But other ports can be mapped to more meaningful components with appropriate options (left) using GDF.

This was achieved by using a GUI definition language, mapping input ports to pre-installed GUI components and as a result showing a much user-friendly interface for workflow execution setup. See Figures 3 and 4 for an example.

This approach offers a great advantage in generating user interfaces, as we have experienced with R GUI Generator (RGG), a software tool which enables the easy generation of graphical user interfaces for the programming language R by adding a few Extensible Markup Language (XML) tags [12].

The Galaxy Project [13] uses a similar approach for local tool integration. But it provides a static description for the tool such as executables, command line syntax, ports, data type and format. However, these description files can only be modified by the server administrator. On the other hand the BIFI approach is a more dynamic, user interface centric approach that complements Taverna Workflow System where users interactively build and exchange information about user interfaces of workflows.

**Figure 5:**
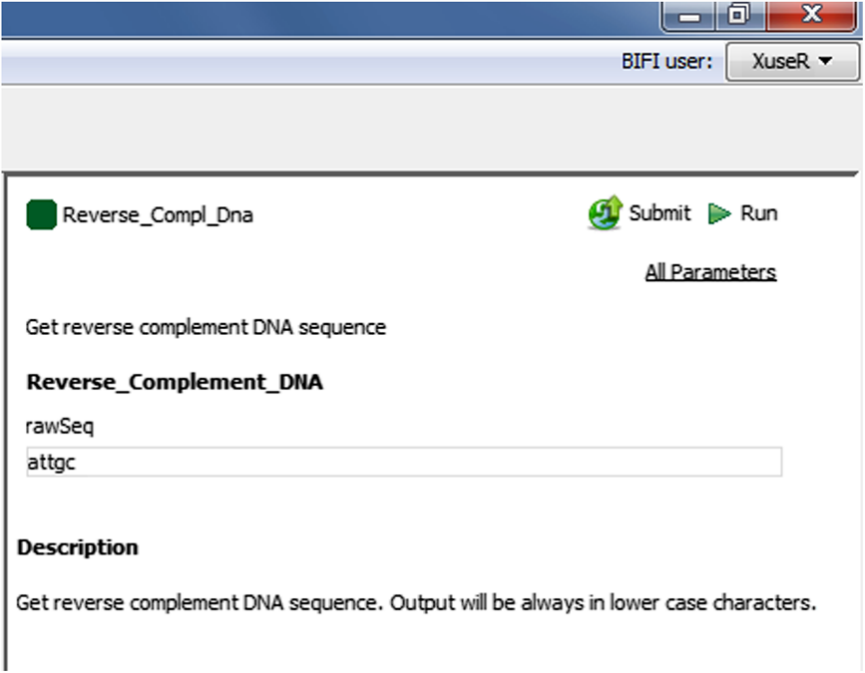
**A simple workflow user interface by BIFI.** A simple workflow user interface generated by BIFI. The GUI description file contains the default values and details about the workflow. With example values already set, the workflow is ready to run with a simple click.

GUI descriptions may include meta information about what kind of data the service is waiting as input or parameter, restrictions and default/example values. A workflow with GDF is therefore perfectly suitable for ready-to-run sample workflow arrangements (see Figure 5). Please see Additional file 2 for the full GDF syntax.

In absence of a GDF instance, we have implemented an algorithm to pick the most appropriate GUI for the port depending on the available meta-information on service descriptions.

## Methods

We have developed a BIFI plugin in Java 1.6 for the Taverna Workbench 2.4. Due to the diversity of the resources and especially because these resources are usually provided by autonomous third parties, it is hardly possible to implement a solution directly in the resource descriptions. We therefore map input ports to specific, pre-installed GUI-components using the graphical user interface description language we have defined.

A workflow in Taverna may have only one GDF instance in the configured GDF repository. In this system we assume that a workflow – GDF pair represents a *tool.* The workflow and the user interface definition file are linked together using workflow identifiers. Each workflow created in Taverna is automatically assigned a universally unique identifier (UUID [14]) which will be kept unique even throughout the modifications.

**Figure 6:**
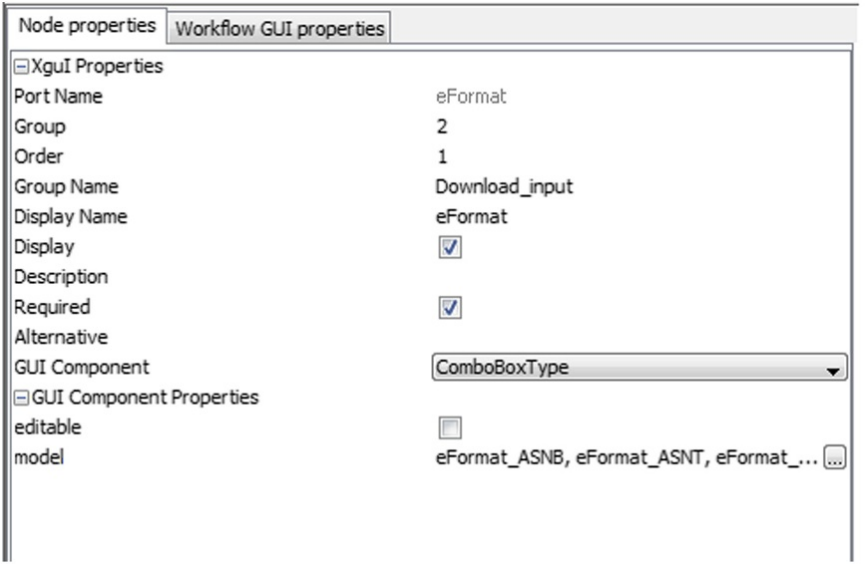
**The BIFI design component.** The BIFI design component allows the user to modify the workflow view or change default values for the ports interactively.

The *Workflow User Interface* (WUI) for the workflow will be initiated immediately after the workflow is opened in the Taverna environment. BIFI searches for the appropriate GDF in the configured repository and renders it. In case of absence of a suitable GDF instance, BIFI generates a default WUI and the corresponding description file using algorithms explained later in this paper. After opening the WUI, users may modify the workflow view or change default values for the ports interactively using the BIFI design component (see Figure 6).

Once the modification is complete, it is possible to submit the instance to the installed repositories using the *Submit* button at the top of the WUI component. If user wants to submit a GDF to the public repository should first login or register. This ensures building a collective experience for better and easier analysis.

**Figure 7:**
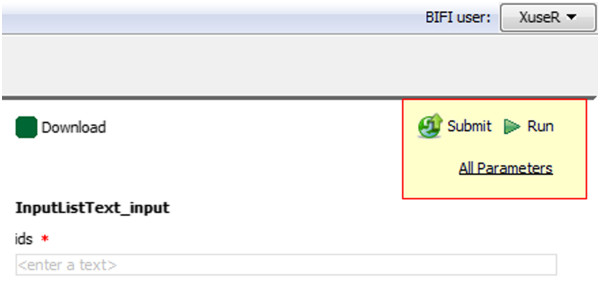
**Buttons of BIFI workflow user interface.** BIFI provides tree buttons on top of WUI. Using the *all parameters* button users can enable all the ports in the workflow, not only defaults. To start execution using the BIFI Plugin inputs, users should use *Run* button provided by the BIFI Plugin. The *Submit* button shows the dialog to submit user interface options to the repository.

To run a workflow using BIFI inputs, users should use the BIFI *Run* button placed on the top right corner of the WUI component (see Figure 7 for BIFI buttons).

### Port mapping

In Taverna each processor has its own input ports. Moreover, workflows should define their own WIPs which again are linked to specific *processor input ports*. As discussed earlier, users execute a workflow by serving the data to the WIPs which are then routed to the defined processor.

**Figure 8:**
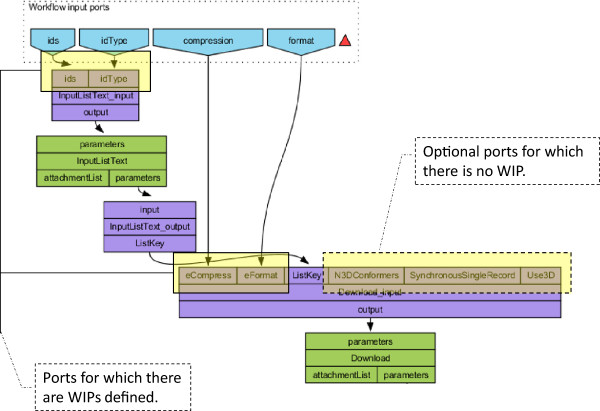
**The first part of the workflow graph for Example Workflow 1.** Some of the processor input ports have WIPs linked to them, some of them have not.

As an example Figure 8 shows a portion of our *Example Workflow 1*. The upper nodes - *ids, idType, compression* and *format* - are WIPs. The former two WIPs serve user data to input ports *ids* and *idType* of the processor *InputListText_input* and the latter two WIPs serve data to the *eCompress* and *eFormat* input ports of the processor *Download_input*.

Different types of services tend to have lots of input ports for configurations, default values and data slots. WIPs enable workflow developers to filter the required ports for successful workflow execution from the rest. As seen in Figure 8, the *N3Dconformers*, *SynchronousSingleRecord* and *Use3D* input ports of the processor *Download_input* are optional for the service and there are no WIPs defined for them. If workflow developers do not define WIPs for a port, however, users will have no possibility to use them even if they wanted to change some optional properties unless they knowingly add WIPs for it. Thus, WIPs bring one more level of complexity for users without an IT background or workflow design expertise.

Once installed, the BIFI plugin changes this approach and enables all the *linkable processor input ports* and ignores WIPs while keeping the ability to filter the required ports. *Linkable ports* are ports having no statically declared value and ports that have no incoming data link from an upstream processor output port. This way, users can use all the linkable ports throughout the workflow without the need for any additional action.

To hide the complexity of non-required ports, BIFI by default shows only the ports for which there is a defined data link from a WIP or the ports, which are configured to be shown in the GDF. This is reasonable because the ports linked to a WIP are presumably important for successful processor execution. Even if not all ports are shown by default, it is always possible to display all the options via the *all parameters* button in the top right corner of the WUI component (see Figure 7).

Before starting the execution, the BIFI adjusts the workflow and creates missing WIPs on the fly and invokes Taverna Execution Engine.

### Algorithm to pick the best user interface component for a port

Our BIFI algorithm enables users to use workflows in an easy and straightforward way even if there is no GUI definition in the configured repository. This is only possible if the data type of ports to be used is predictable and reasonable user interface components for the ports can be programmatically determined.

**Figure 9:**
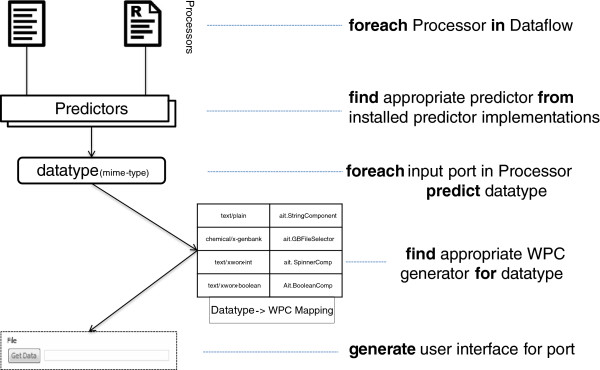
**Mime-type to user interface mapping.** Predictors determine which UI component to use for a specific port by using data from processors and predicting the corresponding data types (mime type). BIFI looks for the default UI for the given data type in the mapping table and creates it for the given port.

As a proof-of-concept implementation, we provide predictors for two Taverna processors: WSDL and R. Predictor implementations are responsible for providing data types and additional information about processor input ports by examining the processors. Figure 9 shows the flow of automatic user interface generation for a specific input port.

**Figure 10:**
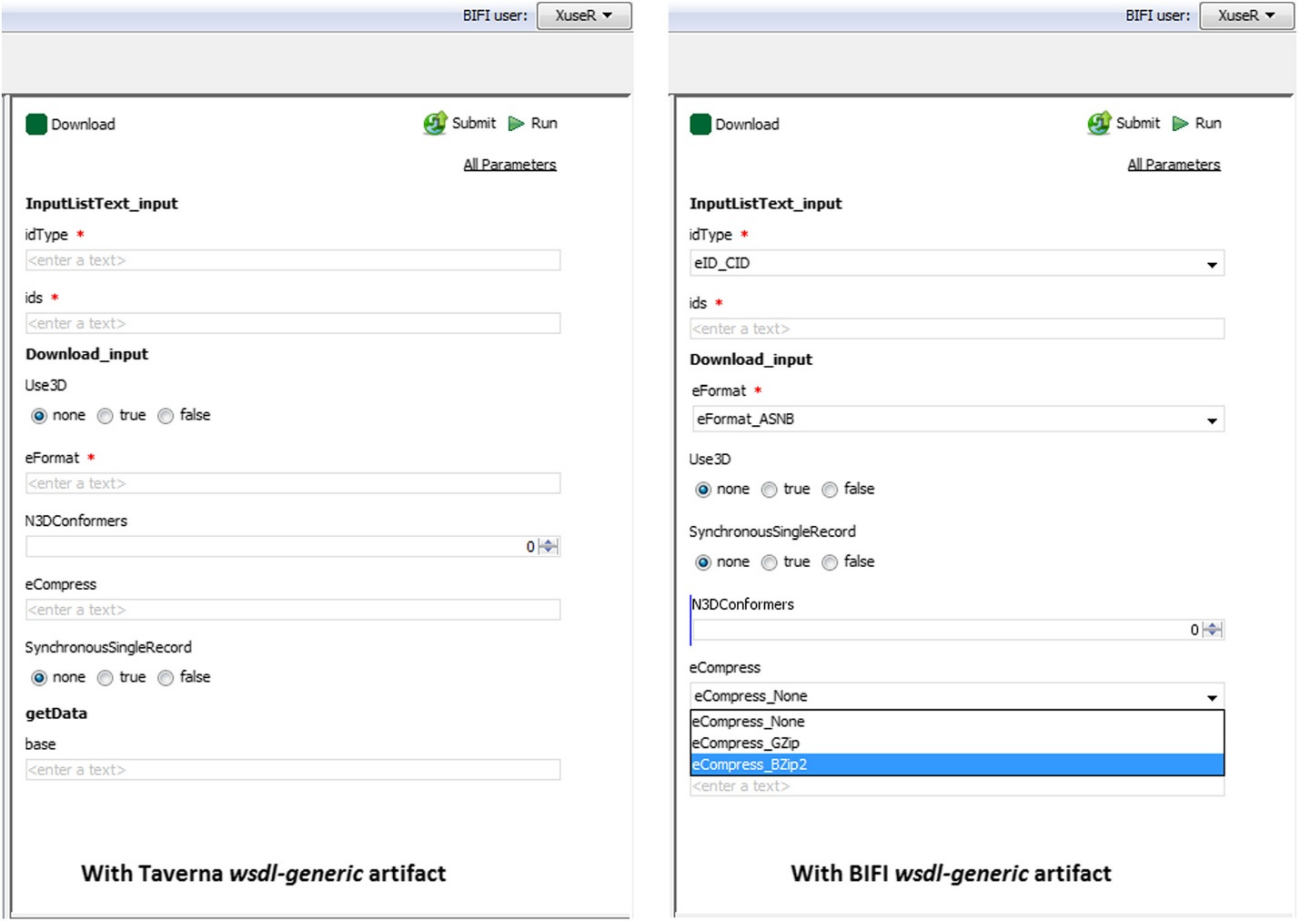
**Comparison of WUIs of example Workflow 1 automatically built using wsdl-artifact provided by the Taverna Workbench and the BIFI Plugin.** BIFI generated workflow user interface for setting input data for Example Workflow 1 (left) using Taverna *wsdl-generic* artifact and (right) using the extended *BIFI wsdl-generic* artifact.

Although enrichment of web service description files with appropriate meta-information about input data is not a common practice, some description files contain such information. However, Taverna completely ignores XSD type information in description files, except base types. We have extended one of the Taverna core modules to extract more information from these files. It is thus only possible to take full advantage of WSDL processor predictors by replacing the original *wsdl-generic* artifact with the extended version we provide^a^. Figure 10 shows a comparison of user interfaces auto-generated using the Taverna version of the *wsdl-generic* artifact and the corresponding BIFI version.

Unlike the WSDL processor, the R processor has no description file to parse. But workflow developers may set the following built-in data types for R processor input ports: *Logical, Numeric, Integer, String, Logical Vector, Numeric Vector, Integer Vector, String Vector, Text-File.* The R predictor uses this information for determining which user interface to use.

Furthermore, BIFI provides the required extension points over service provider interfaces [15] by which developers can add their own *Workflow Port Component* (WPC) implementations as well as data type to WPC generator mappings. Given the properties (like simple type restrictions in XSD), generators create appropriate WPC objects to be shown in the workflow user interface.

## Results

The XworX BIFI plugin brings great simplicity in the usage of the Taverna Workbench. To demonstrate these advantages, the differences between WSDL and R are highlighted using two workflows.

The first workflow consumes SOAP based web services from NCBI PubChem [16] and given a list key, prepares a file containing those records in the selected format for download [17]. The second workflow demonstrates usage of an R package for quality control and primary analysis of Illumina Infinium methylation data [18].

When using Taverna without BIFI, users must enter the workflow inputs for *Example Workflow 1* using the pop-up dialog as shown in Figure 2. The same workflow opened with BIFI enabled Taverna will display a user interface as shown in Figure 10. All available input ports are directly accessible through the BIFI generated GUI.

For the *Example Workflow 2.2* supplied as Additional file 3, BIFI finds an appropriate GDF in the repository and generates the view rendering the GDF. However, for the *Example Workflow 2.1* supplied as Additional file 4, there is no appropriate GDF in the repository. Input data for *Workflow 2.1* and *Workflow 2.2* has been supplied as Additional file 5. Figure 4 shows the difference between the auto-generated view and the view generated using the GUI definition file prepared and submitted to the repository by the workflow creator.

Despite entering data in the string form via the standard Taverna interface, users can use combo boxes, radio buttons with appropriate options or spinners for integer data types. As a result of all this, users are provided with easy-to-use, user-friendly interfaces.

Each port component has its own properties defining the valid data range. Depending on the implementation of the user interface component, it is possible to give instant feedback to the user about the validity of the input data.

Developers are able to implement their own UIs for mime types and install them in Taverna using flexible interfaces provided by Taverna and BIFI. Default properties for given UIs may be overwritten using the *Properties* component (see Figure 6). Users also have the possibility to set preferred installed user interfaces for certain mime types using the *Preferences* window.

Providing intelligent user interfaces ensures that users enjoy easy-to-use software with fewer errors. As a result, users having found that expectations are met will continue using the software environment.

Besides adding more user interface components for different mime types in the future, we plan to add more intelligence to the system to improve the level of automation. Evaluating usage data and inspecting present GDFs in the repository would enable us to infer new information about the mime type to UI component mappings and/or even about the type of the data that should be fed to a certain processor.

## Availability and requirements

**Project name**: The XworX BIFI Plugin for Taverna

**Project home page**: http://www.xworx.org/#!bifi-plugin/c1tou

**Operating system(s):** All major platforms, such as Linux, Windows and Mac OS X

**Programming language**: Java

**Other requirements**: Taverna 2.4, Java 1.6

**License**: LGPL

**Restrictions to use by non-academics**: None

## Electronic supplementary material

Additional file 1: **Example workflow 1.** t2flow format to be opened with Taverna 2.4.1. (ZIP 5 KB)

Additional file 2: **GDF Schema.** GUI definition syntax used by the BIFI Plugin. (ZIP 902 bytes)

Additional file 3: **Example workflow 2_2.** t2flow format to be opened with Taverna 2.4.1. Taverna with BIFI plugin generates automatically a user interface by parsing GDF instance found in the repository. (ZIP 7 KB)

Additional file 4: **Example workflow 2_1.** t2flow format to be opened with Taverna 2.4.1. Taverna with BIFI plugin generates automatically a user interface by using processor input ports data types. For this workflow there is no GDF instance in the repository which BIFI can utilize. (ZIP 7 KB)

Additional file 5: **Input Data for Example workflow 2_X.** The data is taken from official *HumMeth27QCReport* page (http://biocore.crg.cat/wiki/HumMeth27QCReport). (ZIP 2 MB)

## Abbreviations

BIFI: Beautiful interfaces for inputs
WUI: Workflow user interface
GDF: Graphical user interface definition file
WIP: Workflow input port
UI: User interface
WPC: Workflow port component
WDSL: Web Services Description Language.

## References

[1] Li P, Castrillo JI, Velarde G, Wassink I, Soiland-Reyes S, Owen S, Withers D, Oinn T, Pocock MR, Goble C, Oliver SG, Kell DB (2008). Performing statistical analyses on quantitative data in Taverna workflows: an example using R and maxdBrowse to identify differentially-expressed genes from microarray data. BMC Bioinformatics.

[2] Hull D, Wolstencroft K, Stevens R, Goble C, Pocock MR, Li P, Oinn T (2006). Taverna: a tool for building and running workflows of services. Nucleic Acids Res.

[3] **Web Services Activity Statement**. [http://www.w3.org/2002/ws/Activity]

[4] **Web Services Description Language (WSDL) Version 2.0 Part 1: Core Language**. http://www.w3.org/TR/wsdl20/

[5] Wilkinson MD, Links M (2002). BioMOBY: an open source biological web services proposal. Brief Bioinformatics.

[6] Senger M, Rice P, Oinn T (2003). Soaplab - A Unified Sesame Door To Analysis Tools. All Hands Meeting.

[7] **BeanShell - Lightweight Scripting for Java**. [http://www.beanshell.org/home.html]

[8] Wassink I, Rauwerda H, Neerincx PB, Vet PE, Breit TM, Leunissen JA, Nijholt A (2009). Using R in Taverna: RShell v1.2. BMC Res Notes.

[9] **myGrid » Home**. [http://www.mygrid.org.uk/]

[10] **Taverna - open source and domain independent Workflow Management System**. http://www.taverna.org.uk/

[11] Hartmann M (2009). Challenges in Developing User-Adaptive Intelligent User Interfaces. Proceedings of the 17th Workshop on Adaptivity and User Modeling in Interactive Systems at Lernen Wissen Adaptivit (LWA).

[12] Visne I, Dilaveroglu E, Vierlinger K, Lauss M, Yildiz A, Weinhaeusel A, Noehammer C, Leisch F, Kriegner A (2009). RGG: a general GUI Framework for R scripts. BMC Bioinformatics.

[13] Goecks J, Nekrutenko A, Taylor J, Galaxy Team T (2010). Galaxy: a comprehensive approach for supporting accessible, reproducible, and transparent computational research in the life sciences. Genome Biol.

[14] **Universally Unique IDentifier (UUID)**. http://www.ietf.org/rfc/rfc4122.txt

[15] Seacord R, Wrage L (2002). Replaceable Components and the Service Provider Interface.

[16] **The PubChem Project**. http://pubchem.ncbi.nlm.nih.gov/

[17] **myExperiment - Workflows - download (Peter Li) [Taverna 2 Workflow]**. http://www.myexperiment.org/workflows/1435.html

[18] Mancuso FM, Montfort M, Carreras A, Alibes A, Roma G (2011). HumMeth27QCReport: an R package for quality control and primary analysis of Illumina Infinium methylation data. BMC Res Notes.

